# Association of substance use with stress-related sleep disturbance among adolescents in 76 countries: a global population-based study

**DOI:** 10.7189/jogh.15.04195

**Published:** 2025-06-27

**Authors:** Liuqing Li, Zeyan Chen, Danyi Huang, Fei Li, Mengna Pan, Yongliang Zhu, Chuanwei Ma, Jiahong Sun

**Affiliations:** 1 Department of Preventive Medicine, School of Public Health, The First Dongguan Affiliated Hospital, Guangdong Medical University, Dongguan, China; 2 Department of Epidemiology and Health Statistics, School of Public Health, The First Dongguan Affiliated Hospital, Guangdong Medical University, Dongguan, China; 3 Department of Children and Wellness, The First Dongguan Affiliated Hospital, Guangdong Medical University, Dongguan, China

## Abstract

**Background:**

Stress-related sleep disturbance has emerged as a significant public health concern among adolescents worldwide. The independent and combined effects of substance use on stress-related sleep disturbance remain inconclusive. We aimed to explore the association of the use of substances such as tobacco and alcohol with stress-related sleep disturbance among adolescents in 76 countries.

**Methods:**

We collected data from the global school-based student health survey, which comprised 302 181 adolescents aged 12–17 years from 76 countries. The frequency of tobacco and alcohol use in the past 30 days was categorised as follows: zero, one to two, three to nine, 10–29, and 30 days. Tobacco and alcohol use were classified into four categories: non-use, tobacco use alone, alcohol use alone, and combined use. We used multivariate logistic regression analyses to examine the independent and combined associations of tobacco and alcohol use with stress-related sleep disturbance.

**Results:**

As the frequency of tobacco use and alcohol use increases, the proportion of stress-related sleep disturbance among adolescents shows an upward trend (for tobacco use 8.3–27.4%, for alcohol use 6.7–28.9%). Compared to non-drinkers, the odds of having stress-related sleep disturbance increased with frequency of drinking from one to two days to 30 days (from odds ratio (OR) = 1.53; 95% confidence interval (CI) = 1.31–1.78 to OR = 3.13; 95% CI = 1.99–4.90), as well as with the frequency of tobacco use (from OR = 1.11; 95% CI = 0.88–1.39 to OR = 1.98; 95% CI = 1.39–2.81) during the past 30 days.

**Conclusions:**

We found both tobacco and alcohol use, as well as their combination, were associated with stress-related sleep disturbance. These findings emphasise the need to strengthen the prevention and control of tobacco and alcohol use among adolescents in order to reduce stress-related sleep disturbance and improve sleep quality.

Sleep disturbances have become a public health issue among adolescents worldwide, particularly among those with poor mental health status [1]. Data from the global school-based student health survey (GSHS) across 82 countries between 2003–15 revealed that the pooled prevalence of stress-related sleep disturbance among adolescents aged 12–17 years was 9.0%, highest in the Eastern Mediterranean region (17.0%) and lowest in the European region (4.0%) [2]. Sleep disturbance poses a threat not only to growth, learning, and cognitive development [3], but may also be associated with cardiovascular risk in adolescents [4].

Tobacco and alcohol are two common substances used by adolescents. A meta-analysis including 124 554 adolescents showed that among adolescents who used alcohol and tobacco, the prevalence of sleep disturbance was 29% and 28%, respectively [5]. Although several studies have indicated an association between substance use and difficulty initiating sleep among adolescents, the findings remain inconclusive. Bartel and Sancho’s study found that smoking and alcohol consumption were positively associated with sleep disturbance [6,7], however, in Huang’s study, weekly drinking was negatively associated with difficulty initiating sleep (DIS) and early morning awakening (EMA) [8], and Mak’s study found that current smokers, compared with never smokers, were less likely to report DIS (odds ratio (OR) = 0.43; 95% confidence interval (CI) = 0.38–0.50, *P* < 0.001) and EMA (OR = 0.83; 95% CI = 0.73–0.94, *P* = 0.003) [9]. A portion of previous relevant research has focused on the effects of a single alcohol use or tobacco use on sleep disturbance [10–13], or has focused on the effects of smoking and drinking on sleep disturbance without specifically examining the dose-response relationship [6,14–16]. For example, in Lopez-Gil and colleagues’ study, substance use during the past 30 days was categorised into two groups – zero days per month and at least one day per month. Consequently, this classification only allows for exploration of whether alcohol and tobacco use affect sleep disturbance, rather than elucidating the relationship between the dose of substance use and sleep disturbance.

In epidemiological research, the relationship between exposure and effect is one of the core contents. By studying the dose-response relationship, we can more accurately assess the impact of exposure factors on health. Based on this, we hypothesise that there is a dose-response relationship between the frequency of tobacco use and alcohol use and sleep disturbance among students, that is, the higher the frequency of tobacco use and alcohol use, the higher the risk of sleep disturbance. Therefore, in this study, we used data from GSHS between 2009–19 to examine the independent and combined associations of tobacco use and alcohol use with stress-related sleep disturbance among adolescents. We also fill this research gap and provide a scientific basis for the formulation of public health policies by assessing the ‘dose-response’ associations between different tobacco use and alcohol use frequencies and sleep disturbance, and further quantitatively analysing the relationships between different tobacco use and alcohol use frequencies and sleep disturbance.

## METHODS

We derived data from the cross-sectional, population-based GSHS, targeting school-attending children and adolescents aged 12–17 years, conducted across 76 countries between 2009–19. The GSHS employed a consistent two-stage sampling strategy in all participating countries. In the first stage, schools were randomly selected, followed by the random selection of classes from the chosen schools in the second stage. All students from the selected classes were eligible and invited to fill out a standardised and anonymous questionnaire. All participants were volunteers in each country, with written or verbal consent obtained from the adolescents or their parents or guardians. The institutional ethical review board or the national government administrative body approved the GSHS in each participating country. Further details regarding the GSHS can be accessed on the websites of the USA Centres for Disease Control and Prevention and the World Health Organisation (WHO).

### Definition of stress-related sleep disturbance

Respondents were queried with the following question: ‘During the past 12 months, how often have you been so worried about something that you could not sleep at night?’ The response options provided were: ‘never,’ ‘rarely,’ ‘sometimes,’ ‘most of the time,’ and ‘always.’ We categorised adolescents who responded ‘never,’ ‘rarely,’ and ‘sometimes’ as not experiencing stress-related sleep disturbance. Conversely, we categorised those who responded ‘most of the times’ and ‘always’ as experiencing stress-related sleep disturbance.

### Definition of tobacco and alcohol use

The frequency of alcohol use was measured by the question ‘During the past 30 days, on how many days did you have at least one drink containing alcohol?’ with response options of zero, one or two, three to five, six to nine, 10–19, 20–29 days, and every day. Alcohol use was defined as drinking containing alcohol at least one day during the past 30 days. The frequency of tobacco use was measured using two questions: ‘During the past 30 days, on how many days did you smoke cigarettes?’ and ‘During the past 30 days, on how many days did you use any tobacco products other than cigarettes, such as a pipe, rolled tobacco leaves, snuff, or chewing tobacco?’ with response options of zero, one or two, three to five, six to nine, 10–19, 20–29 days, and every day. Tobacco use was defined as the use of cigarettes or any other tobacco products on at least one day during the previous 30 days. Based on the sample distribution, we combined three to five days and six to nine days as three to nine days. Also, we combined 10–19 days and 20–29 days as 10–29 days.

### Covariates

The covariates included in this study include gender, age, consumption of fast food, soft drinks, and fruit/vegetables, how many days bullied past 30 days, days active ≥60 minutes in the past seven days, purchasing power parity per capita (PPP), as well as the World Bank income classification for each country in the survey year, and the survey year. More details can be accessed in the GSHS data repository. It should be noted that the PPP data for the survey year were obtained from the World Bank's official website for all participating countries.

### Statistical analysis

We used the primary sampling unit, stratification, and original sampling weights to calculate the prevalence of tobacco use, alcohol use, and stress-related sleep disturbance in each participating country. We recalculated the sampling weights based on the sample size for each country to derive overall rates and subgroup rates of stress-related sleep disturbance. We used linear trend tests to assess trends in the proportion of adolescents experiencing stress-related sleep disturbance with increasing frequency of tobacco and alcohol use. We used multivariate logistic regression models to assess the ORs and CIs for independent and combined associations of tobacco and alcohol use with stress-related sleep disturbance, adjusting for age, sex, intake of fast food, soft drinks, and fruits/vegetables, how many days bullied past 30 days, days active ≥60 minutes in the past seven days, PPP, World Bank income, and survey year. In addition, we conducted subgroup analyses according to age (12–14 and 15–17 years), sex (male and female), World Bank income (low income, lower-middle income, upper-middle income, and high-income levels), and WHO region (Africa, the Americas, South-East Asia, and the Western Pacific). We considered non-overlapping 95% CIs indicative of statistical differences between groups. We used formal interaction tests (*P*-value for interaction) to validate the gender and age differences. We also conducted a sensitivity analysis in which we excluded the outliers (Tokelau and Afghanistan) and defined alternative sleep disturbance cutoffs (responses of ‘sometimes,’ ‘most of the time,’ and ‘always’) as stress-related sleep disturbance. We performed all statistical analyses using SPSS, version 27.0 (IBM, Armonk, New York, USA). We considered *P*-values <0.05 statistically significant.

## RESULTS

### Characteristics of included participants

A total of 302 181 adolescents (47.4% males) aged 12–17 years from 76 countries in five WHO regions were included in the study (11 in Africa, 25 in the Americas, 13 in the Eastern Mediterranean, nine in South-East Asia, and 18 in the Western Pacific). Overall, the prevalence of current tobacco use was 14.7%, ranging from 3.7% in Cambodia to 53.9% in Tokelau. The prevalence of current alcohol use was 21.5%, ranging from 1.7% in Bangladesh to 54.0% in Argentina. The prevalence of stress-related sleep disturbance was 11.1%, ranging from 3.6% in Myanmar to 22.9% in Afghanistan (Table S1 in the **Online Supplementary Document**).

### Change in proportions of stress-related sleep disturbance with frequency of tobacco or alcohol use

Overall, the proportion of stress-related sleep disturbance exhibited an upward trend with increasing frequency of tobacco use during the past 30 days (zero days: 8.3%, 95% CI = 7.9–8.7; one to two days: 12.5%, 95% CI = 11.0–14.2; three to nine days: 18.1%, 95% CI = 16.1–20.3; 10–29 days: 19.7%, 95% CI = 15.9–24.1; 30 days: 27.4%, 95% CI = 23.2–32.0). Subgroup analyses stratified by gender, age, World Bank income classification, and WHO region revealed similar findings (**Table 1**). The proportion of stress-related sleep disturbance also showed similar results with the increasing frequency of alcohol use during the past 30 days (zero days: 6.7%, 95% CI = 6.3–7.2; one to two days: 12.4%, 95% CI = 11.4–13.4; three to nine days: 18.8%, 95% CI = 16.5–21.3; 10–29 days: 21.8%, 95% CI = 17.0–27.4; 30 days: 28.9%, 95% CI = 22.9–35.8). Subgroup analyses stratified by gender, age, World Bank income classification, and WHO region revealed similar findings (**Table 2**).

**Table 1:** Proportions of stress-related sleep disturbance among adolescents by tobacco use days during the past 30 d*

Group	Countries (n)	Tobacco use days during the past 30 d in days, % (95% CI)					*P value*
		**0**	**1–2**	**3–9**	**10–29**	**30**	
Total	68	8.3 (7.9–8.7)	12.5 (11.0–14.2)	18.1 (16.1–20.3)	19.7 (15.9–24.1)	27.4 (23.2–32.0)	<0.001
Sex							
*Male*	68	6.9 (6.4–7.5)	8.9 (7.3–10.7)	15.8 (13.6–18.4)	16.1 (12.1–21.2)	24.2 (19.5–29.7)	<0.001
*Female*	68	9.7 (9.2–10.3)	20.4 (17.9–23.1)	23.5 (19.6–27.8)	32.0 (25.6–39.1)	41.8 (33.6–50.5)	<0.001
Age group in years							
*12–14*	68	6.9 (6.4–7.3)	11.8 (10.1–13.6)	16.7 (14.1–19.7)	22.2 (18.3–26.8)	32.1 (23.5–42.2)	<0.001
*15–17*	68	10.1 (9.5–10.7)	13.1 (11.0–15.7)	19.3 (16.6–22.3)	18.3 (13.2–24.7)	24.9 (20.8–29.4)	<0.001
World Bank income group							
*Low*	8	8.8 (8.0–9.6)	13.9 (10.1–18.8)	21.8 (16.3–28.7)	22.5 (15.3–31.7)	30.9 (19.5–45.3)	<0.001
*Lower-middle*	20	7.8 (7.2–8.3)	11.0 (9.0–13.4)	17.1 (14.3–20.4)	17.9 (12.7–24.6)	24.7 (18.8–31.8)	<0.001
*Upper-middle*	21	9.0 (8.1–10.0)	15.1 (12.5–18.2)	18.2 (15.5–21.2)	22.4 (16.8–29.2)	31.4 (24.8–38.9)	<0.001
*High*	19	12.3 (11.4–13.1)	16.2 (13.5–19.3)	20.4 (18.0–23.0)	25.2 (21.2–29.6)	24.2 (21.1–27.6)	<0.001
WHO region							
*Africa*	10	9.7 (8.8–10.8)	15.2 (11.7–19.6)	20.9 (16.4–26.2)	28.6 (22.4–35.6)	27.4 (19.8–36.6)	<0.001
*Americas*	19	8.1 (7.5–8.9)	12.1 (9.9–14.7)	14.4 (11.8–17.5)	16.9 (11.9–23.5)	31.1 (23.0–40.4)	<0.001
*Eastern Mediterranean*	13	12.5 (11.5–13.6)	18.3 (15.4–21.5)	22.5 (19.3–26.0)	27.6 (22.2–33.7)	36.7 (30.1–43.8)	<0.001
*South-East Asia*	8	4.4 (3.9–5.0)	5.9 (3.8–9.1)	16.5 (12.4–21.6)	12.7 (7.0–22.0)	20.3 (11.8–32.6)	<0.001
*Western Pacific*	18	8.5 (7.9–9.2)	9.9 (7.8–12.4)	10.9 (6.9–16.8)	14.9 (10.9–20.1)	18.0 (13.7–23.4)	<0.001

CI – confidence interval, WHO – World Health Organization

**Table 2:** Proportions of stress-related sleep disturbance among adolescents by alcohol use days during the past 30 d

Group	Countries (n)	Alcohol use days during the past 30 d in days, % (95% CI)					*P*-value
		**0**	**1–2**	**3–9**	**10–29**	**30**	
Total	63	6.7 (6.3–7.2)	12.4 (11.4–13.4)	18.8 (16.5–21.3)	21.8 (17.0–27.4)	28.9 (22.9–35.8)	<0.001
Sex							
*Males*	63	6.0 (5.4–6.6)	10.2 (8.8–11.7)	14.7 (11.8–18.2)	21.5 (15.0–30.0)	24.1 (17.3–32.5)	<0.001
*Females*	63	7.5 (7.1–7.9)	15.2 (13.8–16.7)	25.4 (21.7–29.4)	22.1 (16.9–28.5)	38.0 (29.1–47.9)	<0.001
Age group in years							
*12–14*	63	5.9 (5.4–6.3)	11.5 (9.9–13.4)	19.5 (16.7–22.7)	23.5 (14.0–36.7)	32.6 (23.8–42.9)	<0.001
*15–17*	63	7.9 (7.3–8.5)	12.9 (11.7–14.2)	18.4 (15.3–22.0)	20.7 (16.3–25.9)	27.2 (19.9–36.0)	<0.001
World Bank income group							
*Low*	8	7.7 (6.9–8.5)	14.0 (11.2–17.4)	20.3 (15.0–27.0)	24.2 (16.4–34.2)	46.9 (29.8–64.7)	<0.001
*Lower-middle*	20	6.2 (5.7–6.8)	11.9 (10.4–13.6)	21.6 (16.8–27.3)	25.0 (15.4–37.7)	26.3 (18.7–35.7)	<0.001
*Upper-middle*	21	7.3 (6.7–7.9)	12.2 (10.8–13.8)	15.9 (13.7–18.5)	18.4 (13.5–24.5)	23.1 (14.9–33.9)	<0.001
*High*	14	9.1 (8.5–9.8)	11.8 (10.6–13.2)	16.0 (13.6–18.8)	16.6 (13.4–20.3)	23.0 (16.1–31.6)	<0.001
WHO region							
*Africa*	10	9.3 (8.3–10.3)	14.9 (12.4–17.8)	23.2 (18.1–29.3)	27.7 (20.8–35.8)	46.0 (32.0–60.6)	<0.001
*Americas*	25	6.8 (6.4–7.3)	12.2 (10.9–13.6)	15.4 (13.4–17.6)	19.4 (14.6–25.3)	18.6 (12.6–26.7)	<0.001
*Eastern Mediterranean*	2	14.4 (12.3–16.7)	15.3 (9.6–23.4)	23.1 (14.9–33.9)	18.8 (5.2–49.2)	21.2 (7.4–47.6)	0.474
*South-East Asia*	8	4.3 (3.8–4.8)	10.6 (7.6–14.6)	22.3 (14.9–31.8)	20.6 (8.1–43.3)	31.7 (18.3–49.1)	<0.001
*Western Pacific*	18	8.2 (7.5–8.8)	10.8 (9.3–12.6)	15.6 (11.7–20.7)	20.4 (13.5–29.5)	30.9 (20.0–44.5)	<0.001

CI – confidence interval, WHO – World Health Organization

### Comparing proportions of stress-related sleep disturbance among adolescents: users of both tobacco and alcohol, alcohol users alone, tobacco users alone, and non-users

Overall, the proportion of stress-related sleep disturbance was highest among adolescents who use both tobacco and alcohol (19.3%), followed by those who use alcohol alone (13.1%), those who use tobacco alone (11.4%), and those who use neither tobacco nor alcohol (6.5%). Subgroup analyses stratified by gender, age, World Bank income classification, and WHO region revealed similar findings (Table S2 in the **Online Supplementary Document**).

### Change in odds of stress-related sleep disturbance with the frequency of tobacco or alcohol use

As the frequency of tobacco use during the past 30 days increased, the OR values for stress-related sleep disturbance increased from OR = 1.11 (95% CI = 0.88–1.39) for one to two days, to OR = 1.62 (95% CI = 1.23–2.12) for three to nine days, OR = 1.76 (95% CI = 1.18–2.64) for 10–29 days, and OR = 1.98 (95% CI = 1.39–2.81) for 30 days, after adjusting for potential confounders. Similar trends were observed in subgroup analyses stratified by gender, age, World Bank income group, and WHO region (**Table 3**). As the frequency of alcohol use increased, the OR values for stress-related sleep disturbance increased from OR = 1.53 (95% CI = 1.31–1.78) for one to two days, to OR = 1.85 (95% CI = 1.44–2.38) for three to nine days, OR = 2.21 (95% CI = 1.44–3.39) for 10–29 days, and OR = 3.13 (95% CI = 1.99–4.90) for 30 days, after adjusting for potential confounders. Similar trends have been observed in subgroup analyses by gender, age, World Bank income group, and WHO region (Table S3 in the **Online Supplementary Document**). Formal interaction tests showed that the correlation coefficients decreased as the frequency of tobacco/alcohol use subgroups increased, suggesting that the correlation between gender/age diminished as the frequency of tobacco use increased. However, the difference remained significant. This conclusion is consistent with the results determined using confidence intervals. (Table S4–7 in the **Online Supplementary Document**). The sensitivity analyses, excluding outlier countries and redefining stress-related sleep disturbance, showed similar results (Table S8–11 in the **Online Supplementary Document**).

**Table 3:** Association between tobacco use and stress-related sleep disturbance among adolescents by sex, age group, World Bank income group, and WHO region*

Group	Tobacco use days during the past 30 d in days, OR (95%CI)†					*P*-value	
	**1–2**	**3–9**	**10–29**	**≥30**		
Total	1.11 (0.88–1.39)	1.62 (1.23–2.12)	1.76 (1.18–2.64)	1.98 (1.39–2.81)	<0.001	
Sex						
*Males*	0.94 (0.66–1.35)	1.53 (1.05–2.25)	1.74 (1.01–3.02)	1.94 (1.25–3.01)	0.007	
*Females*	1.41 (1.08–1.83)	1.83 (1.28–2.61)	2.03 (1.22–3.36)	2.59 (1.58–4.25)	<0.001	
Age group in years						
*12–14*	1.19 (0.85–1.65)	1.59 (0.94–2.69)	1.90 (1.26–2.86)	2.71 (1.45–5.08)	<0.001	
*15–17*	1.06 (0.79–1.42)	1.65 (1.22–2.22)	1.72 (0.99–2.98)	1.68 (1.14–2.46)	0.016	
World Bank income group						
*Low*	1.53 (0.91–2.55)	1.89 (1.07–3.34)	1.94 (0.90–4.19)	4.29 (1.80–10.21)	0.022	
*Lower-middle*	0.88 (0.60–1.30)	1.48 (0.95–2.31)	1.64 (0.86–3.14)	1.46 (0.85–2.52)	0.126	
*Upper-middle*	1.35 (1.02–1.78)	1.58 (1.19–2.10)	1.83 (1.23–2.72)	2.33 (1.55–3.49)	<0.001	
*High*	1.07 (0.84–1.36)	1.64 (1.30–2.06)	1.65 (1.18–2.29)	1.36 (0.98–1.87)	<0.001	
WHO region						
*Africa*	1.18 (0.76–1.83)	1.52 (0.97–2.36)	2.11 (1.34–3.34)	1.55 (0.82–2.93)	0.032	
*Americas*	1.29 (0.92–1.81)	1.39 (0.97–1.98)	1.56 (0.84–2.87)	3.16 (1.72–5.81)	0.013	
*Eastern Mediterranean*	1.72 (1.34–2.20)	2.06 (1.52–2.79)	1.86 (1.27–2.73)	2.65 (1.88–3.72)	<0.001	
*South-East Asia*	1.03 (0.56–1.90)	2.54 (1.43–4.50)	1.98 (0.87–4.51)	2.46 (1.25–4.82)	0.023	
*Western Pacific*	1.01 (0.73–1.39)	0.97 (0.57–1.63)	1.19 (0.74–1.92)	1.57 (1.01–2.44)	0.364	

CI – confidence interval, OR – odds ratio, WHO – World Health Organization

*Adjusted for sex, age, survey year, intake of fast food, soft drinks, and fruit/vegetables, and World Bank income in the survey year for each country, how many days bullied past 30 d, days active 60 min plus past 7 d, PPP, alcohol use, region.

†Zero days was used as a reference group.

### Comparing odds of stress-related sleep disturbance among adolescents: users of both tobacco and alcohol, alcohol users alone, tobacco users alone

**Figure 1:**
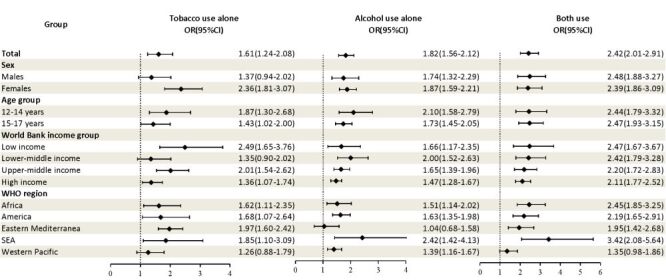
Association of tobacco use alone, alcohol use alone, or combined use of both substances with stress-related sleep disturbance among adolescents by sex, age group, World Bank income group, and WHO region. Adjusted for sex, age, survey year, intake of fast food, soft drinks, and fruit/vegetables, and World Bank income in the survey year for each country, days active ≥60 minutes in the past seven days, how many days bullied in the past 30 days, PPP, region.

Compared with adolescents who used neither tobacco nor alcohol, those who used tobacco and alcohol at the same time (OR = 2.42; 95% CI = 2.01–2.91) were more likely to experience stress-related sleep disturbance than those who used tobacco alone (OR = 1.61; 95% CI = 1.24–2.08) or used alcohol alone (OR = 1.82; 95% CI = 1.56–2.12). Among individuals who used tobacco or alcohol, females, younger adolescents, and those from low-income countries were more likely to have stress-related sleep disturbance compared to males, older adolescents, and those from other countries (**Figure 1**).

## DISCUSSION

We found that substance use, including tobacco and/or alcohol use, was positively associated with stress-related sleep disturbance among adolescents from 76 countries. There was a ‘dose-response’ association between tobacco use or alcohol use and stress-related sleep disturbance. In addition, among adolescents who used tobacco, alcohol, or both, females, older adolescents, and those from low-income countries were more likely to experience stress-related sleep disturbance.

We found that the lowest prevalence of tobacco use was in Cambodia (3.7%) and the highest was in Tokelau (53.9%), with an overall prevalence of 14.7%. The overall prevalence of alcohol use was 21.5%, ranging from 1.7% in Bangladesh to 54.0% in Argentina. Almila and colleagues' study also showed that prevalence varied by region with countries, which may be due to variations in the socio-psycho-cultural environment [17]. We also found that there is a significant difference in the overall prevalence of stress-related sleep disturbance, currently at 11.1%, ranging from 3.6% in Myanmar to 22.9% in Afghanistan, a result consistent with a previous study [18]. This discrepancy may be due to the fact that some countries are not members of the Framework Convention for Tobacco Control [19]. Differences in the prevalence of alcohol use may be due to different alcohol control policies [20]. These findings highlight the need for countries to pay attention to stress-related sleep disturbance and tobacco and alcohol use among adolescents. However, past studies have often been based on small sample sizes or limited to specific regions within countries, and have overlooked the effects of tobacco and alcohol dosage on sleep [15,16]. We used standardised criteria to collect questionnaire data, involved 76 countries worldwide, had a much larger sample size, and provided highly persuasive evidence regarding the current status of the effects of tobacco and alcohol use and stress-related sleep disturbance.

Several previous studies have shown that tobacco use is not significantly associated with sleep disturbance [9,14]. In contrast, other studies have found that alcohol use and tobacco use are only associated with sleep disturbance in women [13,14,16,21]. However, using global data, we found that both smoking and alcohol use were associated with stress-related sleep disturbance, and this is related to gender. Nevertheless, women appear to be more susceptible to the effects of these substances than men, a finding consistent with some other studies [10,17,22,23]. This heightened susceptibility may be due to women being more psychologically sensitive compared to men, leading to increased anxiety in response to life and academic stresses. In the study by Almila and colleagues, it was shown that women were more likely to consume large amounts of alcohol when they were in a bad mood or when they were nervous. However, more specific and specialised experimental investigations are needed for the specific mechanisms. Almila and colleagues' study also suggests that biological differences between men and women may play a role, as women tend to have higher blood alcohol concentrations after consuming the same amount of alcohol [17]. This finding underscores the need to pay more attention to adolescent girls when formulating measures related to substance use, in order to ensure their healthier development. For example, parents can be provided with training on relevant psychological knowledge, and schools can conduct synchronised regular screening of students' emotional problems, drinking and stress-related sleep disturbance among adolescent girls due to life or school stress.

In other studies, they find a tendency for older adolescents to have a higher risk of sleep disturbance [22,24,25], while in our study, we observed that younger adolescents have a higher risk of stress-related sleep disturbance. However, the trend we observed is consistent with much of the research on anxiety-related disorders: younger people are more likely to be anxious. The possible reason is that, compared with older adolescents, younger adolescents are more likely to experience symptoms such as stress-related sleep disturbance and are more vulnerable when it comes to resisting the use of harmful substances like smoking and drinking. In addition, context-specific and methodological variations in early studies may account for these inconsistencies [22]. This may be due to the fact that younger adolescents are less mature in their level of psychological development and are more susceptible to the effects of, for example, academic pressures. Similarly, while there was no significant difference in terms of region, we observed that adolescents from low-income countries tended to have a higher risk of stress-related sleep disturbance. This could be attributed to poverty and limited access to resources for addressing anxiety [26,27]. All of these factors are related to psychological problems and should be considered as potential targets for interventions aimed at minimising the occurrence of anxiety. These findings underscore the need to fully consider the economic levels of different countries and the age of adolescents when formulating protective measures against stress-related sleep disturbances among adolescents, in order to achieve more precise prevention and control of stress-related sleep disturbances among this population. For example, legislation should be passed to further restrict the age of purchase of tobacco and alcohol, to increase the regulation of underage purchases of tobacco and alcohol, and to increase education in school curricula on the physical and mental health effects of tobacco and alcohol consumption.

The reasons that substance use exacerbates stress-related sleep problems may be attributed to the physiological effects of alcohol and nicotine on sleep architecture and stress response systems. Mak and colleagues' research indicates that adolescent smokers, both experimental and current, are more likely to report snoring and difficulty breathing during sleep compared to never-smokers [9]. This suggests that the physiological impact of nicotine may disrupt normal respiratory patterns during sleep, leading to increased instances of snoring and breathing difficulties. Similarly, Vitiello and colleagues' research suggests that while alcohol is only a mild respiratory depressant in the waking state, a substantial body of literature now indicates that pre-bedtime alcohol consumption can induce sleep-related breathing disturbance or exacerbate pre-existing obstructive sleep apnea [28]. The consumption of alcohol before bedtime may relax the muscles in the throat, leading to a narrowing of the airway and increased resistance to airflow, which can result in snoring and other breathing-related sleep disturbances. These findings highlight the complex interplay between substance use and sleep disturbance, emphasising the need for a comprehensive understanding of the physiological mechanisms involved. Future research should continue to explore the specific ways in which alcohol and nicotine influence sleep architecture and stress response systems to more accurately assess causality by tracking adolescents’ tobacco and alcohol use behaviours over time and observing how they influence the onset and progression of stress-related sleep disturbance.

### Strengths and limitations

We utilised relevant data from 76 countries collected by GSHS, all collected using uniform standards, and the large sample size ensured the reliability of the results. However, despite these strengths, our study has some limitations. First, the self-reported nature of data may introduce bias, including social desirability bias and recall bias. Second, as a cross-sectional study, our findings cannot establish a causal relationship between tobacco and/or alcohol use and stress-related sleep disturbance. Prospective cohort studies are needed to explore this association further. Third, GSHS data collection was conducted in schools, which limited our sample to school-attending adolescents and potentially excluded those not in formal education settings. Fourth, the definition of stress-related sleep disturbance in this study was based on the content of the questionnaire, ‘During the past 12 months, how often have you been so worried about something that you could not sleep at night?’ rather than the clinical aspects of the diagnosis. Thus, it was not possible to explore the relationship between substance use and more nuanced classifications of sleep disturbance (*e.g.* difficulty falling asleep, early waking, *etc.*), which limited the analysis to a general assessment of alcohol and tobacco use. Fifth, there may also be some confounding factors not accounted for in our study. For example, mental health conditions, family stress, and adverse life events (such as the death of a family member or other significant stressors) could impact both substance use and stress-related sleep disturbance. Sixth, the frequency of tobacco and alcohol use was obtained through questionnaires, and it was not possible to determine the exact amount of tobacco and alcohol consumed. More accurate assessments of these specific details will be needed in the future. Seventh, substance use also includes marijuana, amphetamines, and others. However, only tobacco and alcohol use were included in this study due to severe relevant sample deficiencies.

## CONCLUSIONS

In conclusion, our study provides robust empirical evidence of a positive association between tobacco and/or alcohol use and stress-related sleep disturbance. The prevalence of these disturbances is significantly associated with several demographic and socioeconomic factors, including age, gender, income, and region. Given these findings, there is an urgent need to develop and implement comprehensive public health strategies aimed at reducing the adverse effects of substance use on adolescent sleep. Such strategies should be tailored to address the specific needs of different demographic groups, taking into account the varying prevalence and impact of stress-related sleep disturbance across age, gender, income, and regional lines. By doing so, we cannot only improve the sleep quality of adolescents but also enhance their overall health and development on a global scale.

## Additional material


Online Supplementary Document
