# Supplementary material for: Association of substance use with stress-related sleep disturbance among adolescents in 76 countries: a global population-based study
Source: J Glob Health. 2025 Jun 27;15:04195. doi: 10.7189/jogh.15.04195 (PMC12203626; doi:10.7189/jogh.15.04195)
Supplement: Online Supplementary Document [file jogh-15-04195-s001.pdf]

Supplement to: Li L, Chen Z, Huang D, Li F, Pan M, Zhu Y, Ma C, Sun J. Association of substance use with stress-related sleep disturbance among adolescents in 76 countries: a global population-based study. J Glob Health. 2025;15:04195.

Table S1. Characteristics of the Global School-based Student Health Survey of adolescents aged 12-17 years in 76 countries (data from 2009-2019)

Country	Income classification	Survey year	Sample size of population	Males, %	Prevalence of current tobacco use, %	Prevalence of current alcohol use, %	prevalence of stress-related sleep disturbance, %	Mean age, years
**Africa**								
Benin	Low	2016	1567	68.8	7.1	41.4	18.2	15.4
Ghana	Lower-middle	2012	2357	50.3	15.3	14.5	13.1	15.1
Liberia	Low	2017	1177	52.2	11.5	18.4	15.8	15.5
Malawi	Low	2009	2197	51.6	7.8	3.8	13.4	13.9
Mauritania	Lower-middle	2010	1932	54.3	23.1	—	11.4	14.8
Mauritius	Upper-middle	2017	2898	46.4	19.6	25.7	9.3	14.8
Mozambique	Low	2015	1263	52.2	4.6	12.3	9.4	15.4
Namibia	Upper-middle	2013	3276	44.6	12.3	29.6	14.3	15.1
Seychelles	High	2015	2451	50.1	23.1	47.5	11.1	13.9
Sierra Leone	Low	2017	2236	50.2	—	12.9	17.8	15.0
Tanzania	Low	2014	3343	49.0	6.2	3.9	5.9	14.3
**Americas**								
Anguilla	High	2016	788	48.5	12.1	32.7	9.7	14.7
Antigua and Barbuda	Upper-middle	2009	1201	51.3	11.5	44.1	14.3	13.9

Argentina	Upper-middle	2018	55,507	47.9	20.0	54.0	13.2	14.9
Bahamas	High	2013	1312	47.8	9.5	27.4	14.1	13.4
Barbados	High	2011	1580	49.1	14.3	46.6	10.1	14.2
Belize	Lower-middle	2011	1875	48.4	—	29.3	12.2	14.0
Bolivia	Lower-middle	2018	6394	49.8	—	23.6	13.0	15.2
British Virgin Islands	High	2009	1573	47.8	8.6	35.9	10.7	14.2
Costa Rica	Upper-middle	2009	2650	50.5	12.8	26.6	5.1	14.2
Curacao	High	2015	2160	49.1	12.3	36.0	10.8	14.7
Dominica	Upper-middle	2009	1495	51.1	—	53.8	9.9	14.0
Dominican Republic	Upper-middle	2016	1319	50.5	10.9	42.6	10.1	14.7
El Salvador	Lower-middle	2013	1780	51.6	—	17.7	7.1	14.3
Guatemala	Lower-middle	2015	4007	52.3	—	17.8	6.5	14.3
Guyana	Lower-middle	2010	2330	48.8	15.1	41.0	14.1	14.4
Honduras	Lower-middle	2012	1704	46.9	13.7	15.9	5.7	14.0
Jamaica	Upper-middle	2017	1582	48.7	18.8	48.9	12.9	15.0
Panama	High	2018	2603	47.1	9.7	28.8	10.1	15.1
Paraguay	Upper-middle	2017	2819	48.7	—	34.3	9.1	14.7
Peru	Upper-middle	2010	2825	50.3	19.4	29.4	8.9	14.5
Saint Lucia	Upper-middle	2018	1809	48.0	9.8	47.1	14.0	14.4
Saint Vincent and the Grenadines	Upper-middle	2018	1684	47.8	11.3	46.4	15.7	15.1
Suriname	Upper-middle	2016	1884	49.0	14.0	38.8	12.3	14.5
Trinidad and Tobago	High	2017	3569	48.1	12.2	29.8	13.7	14.2
Uruguay	High	2012	3400	45.6	13.6	48.5	5.9	14.4
**Eastern Mediterranean**								
Afghanistan	Low	2014	2116	53.4	8.7	—	22.9	14.8
Bahrain	High	2016	6823	50.8	20.3	—	16.2	14.2

Iraq	Upper-middle	2012	1955	57.2	14.7	—	12.9	14.4
Kuwait	High	2015	3128	49.4	26.1	—	20.4	15.0
Lebanon	Upper-middle	2017	5123	46.6	32.9	17.5	13.3	14.7
Morocco	Lower-middle	2016	5640	52.9	11.3	—	16.0	14.5
Oman	High	2015	3094	46.3	7.8	—	18.7	15.3
Pakistan	Lower-middle	2009	5136	49.4	10.1	—	8.4	14.2
Palestine	Lower-middle	2010	13,950	46.8	20.7	—	16.4	13.9
Qatar	High	2011	1676	51.4	30.6	—	18.3	13.5
Syria	Lower-middle	2010	2986	48.0	19.8	7.0	14.9	13.7
United Arab Emirates	High	2016	5306	53.7	16.5	—	15.8	15.8
Yemen	Lower-middle	2014	2158	53.4	15.1	—	14.8	14.6
**South-East Asia**								
Bangladesh	Lower-middle	2014	2957	65.2	9.6	1.7	4.6	14.2
Bhutan	Lower-middle	2016	6020	46.1	28.4	23.5	7.6	15.1
Indonesia	Lower-middle	2015	10,619	48.8	12.3	4.1	4.5	14.0
Maldives	Upper-middle	2014	2929	50.9	11.7	—	14.9	15.2
Myanmar	Lower-middle	2016	2666	45.7	—	4.2	3.6	14.2
Nepal	Low	2015	6122	49.0	7.7	5.0	4.3	14.5
Sri Lanka	Lower-middle	2016	3177	48.5	8.8	3.0	4.6	14.7
Thailand	Upper-middle	2015	5504	46.0	12.7	20.9	8.7	14.4
Timor-Leste	Lower-middle	2015	2877	48.2	26.3	15.0	11.3	15.2
**Western Pacific**								
Brunei Darussalam	High	2019	2286	50.4	16.8	3.4	14.9	14.6
Cambodia	Low	2013	2908	50.1	3.7	7.1	5.1	15.0
Cook Islands	High	2015	647	48.6	21.3	34.5	14.0	15.2
Fiji	Upper-middle	2016	2983	49.0	14.6	16.4	12.4	15.4

French Polynesia	High	2015	2856	50.0	23.7	40.4	11.4	14.7
Kiribati	Lower-middle	2011	1549	47.1	32.6	31.8	9.1	14.3
Laos	Lower-middle	2015	3596	53.1	6.1	29.6	4.9	15.6
Malaysia	Upper-middle	2012	25,154	50.0	12.9	8.6	5.3	14.9
Mongolia	Lower-middle	2013	5093	48.2	11.5	8.1	5.5	14.4
Nauru	Upper-middle	2011	519	46.7	28.8	24.7	16.8	14.5
Philippines	Lower-middle	2015	8308	48.6	15.0	20.4	10.7	14.5
Samoa	Upper-middle	2017	1658	47.2	13.1	11.1	9.4	14.9
Solomon Islands	Lower-middle	2011	1262	54.5	31.4	20.6	13.0	14.7
Tokelau	High	2014	108	56.2	53.9	47.1	10.3	14.2
Tonga	Upper-middle	2017	2828	50.4	20.3	13.2	14.6	14.4
Tuvalu	Upper-middle	2013	891	48.3	21.7	15.7	6.2	14.0
Vanuatu	Lower-middle	2016	2014	49.3	24.8	17.2	6.8	14.9
Wallis and Futuna	High	2015	1012	48.7	30.8	32.3	15.7	14.5
**Total**			302,181	47.4	14.7	21.5	11.1	14.6

Table S2. Proportions (%) of stress-related sleep disturbance among adolescents by tobacco use and alcohol use (≥1 day during the past 30 days)

Group	No. of countries	Neither	Tobacco use alone	Alcohol use alone	Both use
**Total**	55	6.5(6.1-6.9)	11.4(9.6-13.4)	13.1(11.9-14.4)	19.3(17.2-21.6)
**Sex**					
Males	55	5.8(5.1-6.5)	8.8(6.9-11.1)	10.6(9.0-12.5)	16.7(14.1-19.7)
Females	55	7.2(6.8-7.7)	19.6(16.6-23.0)	15.9(14.3-17.7)	24.7(21.2-28.5)
**Age group**					
12-14 years	55	5.7(5.2-6.2)	11.6(9.5-14.1)	12.5(10.4-15.0)	19.1(16.0-22.6)
15-17 years	55	7.6(7.0-8.2)	11.1(8.7-14.2)	13.4(11.9-15.0)	19.5(16.8-22.5)
**World Bank income group**					
Low income	7	6.9(6.2-7.8)	15.6(11.6-20.7)	14.9(11.8-18.6)	23.1(17.9-29.3)
Lower-middle income	15	6.2(5.6-6.8)	10.0(7.9-12.6)	12.7(10.6-15.2)	19.7(16.0-24.0)
Upper-middle income	19	6.8(6.2-7.4)	14.1(11.4-17.2)	12.5(10.9-14.3)	17.5(15.3-20.0)
High income	14	8.7(8.1-9.4)	14.7(12.2-17.6)	12.2(10.9-13.5)	18.2(16.0-20.5)
**WHO region**					
Africa	9	8.7(7.7-9.7)	14.7(11.1-19.3)	15.7(13.0-18.8)	26.6(22.6-31.0)
America	19	6.6(6.0-7.2)	11.5(8.6-15.3)	12.2(10.6-14.0)	16.3(14.1-18.8)
Eastern Mediterranean	2	13.0(10.8-15.6)	20.6(17.9-23.6)	6.9(4.1-11.2)	25.1(17.2-35.0)
SEA	7	4.1(3.6-4.6)	8.2(5.7-11.6)	12.9(9.0-18.1)	20.2(14.8-27.0)
Western Pacific	18	8.0(7.4-8.7)	10.0(7.7-13.0)	11.9(10.3-13.7)	13.1(10.0-17.0)

Data are presented as %(95%CI).

Table S3. Association between alcohol use and stress-related sleep disturbance among adolescents by sex, age group, World Bank income group, and WHO region

Group	Alcohol use days during the past 30 days, OR(95%CI)					*P* for trend
	0 days	1-2 days	3-9days	10-29 days	≥30 days	
**Total**	1.00	1.53(1.31-1.78)	1.85(1.44-2.38)	2.21(1.44-3.39)	3.13(1.99-4.90)	<0.001
**Sex**						
Males	1.00	1.51(1.16-1.96)	1.66(1.10-2.51)	2.75(1.54-4.93)	3.10(1.75-5.48)	<0.001
Females	1.00	1.50(1.27-1.77)	2.02(1.54-2.65)	1.51(0.80-2.83)	3.18(1.46-6.95)	<0.001
**Age group**						
12-14 years	1.00	1.58(1.18-2.12)	1.87(1.22-2.85)	1.75(0.70-4.35)	4.02(1.86-8.67)	<0.001
15-17 years	1.00	1.52(1.27-1.82)	1.89(1.40-2.56)	2.49(1.63-3.81)	2.95(1.71-5.06)	<0.001
**World Bank income group**						
Low income	1.00	1.36(0.96-1.93)	1.42(0.84-2.40)	1.89(0.87-4.10)	6.16(2.04-18.60)	0.008
Lower-middle income	1.00	1.52(1.14-2.02)	2.39(1.49-3.81)	2.66(1.16-6.14)	3.74(2.07-6.77)	<0.001
Upper-middle income	1.00	1.45(1.20-1.76)	1.51(1.18-1.92)	1.96(1.16-3.31)	1.76(0.81-3.82)	<0.001
High income	1.00	1.35(1.16-1.57)	1.75(1.46-2.10)	1.59(1.19-2.13)	1.86(1.08-3.21)	<0.001
**WHO region**						
Africa	1.00	1.24(0.92-1.69)	1.72(1.12-2.64)	2.13(1.31-3.45)	4.73(2.31-9.70)	<0.001
Americas	1.00	1.47(1.19-1.83)	1.50(1.14-1.98)	2.51(1.44-4.38)	1.73(0.79-3.81)	0.002

Eastern Mediterranean	1.00	0.95(0.67-1.34)	0.84(0.55-1.27)	1.38(0.71-2.68)	2.43(0.92-6.44)	0.240
South-East Asia	1.00	1.76(1.04-2.97)	2.46(1.22-4.96)	1.58(0.42-5.94)	2.35(0.66-8.39)	0.034
Western Pacific	1.00	1.20(1.00-1.45)	1.44(0.99-2.11)	2.38(1.27-4.45)	2.84(1.44-5.58)	0.003

Adjusted for sex, age, survey year, intake of fast food, soft drinks, and fruit/vegetables, and World Bank income in the survey year for each country, how many days bullied past 30 days, days active 60 min plus past 7 days, PPP, tobacco use, region

Table S4. Correlation matrix of tobacco use frequency groups by sex, with significance indicators

	**[tobacco group 1] * sex**	**[tobacco group 2] * sex**	**[tobacco group 3] * sex**	**[tobacco group 4] * sex**	**[tobacco group 5] * sex**
**[tobacco group 1] * sex**	1				
**[tobacco group 2] * sex**	0.770*	1			
**[tobacco group 3] * sex**	0.637*	0.544*	1		
**[tobacco group 4] * sex**	0.565*	0.428*	0.386*	1	
**[tobacco group 5] * sex**	0.483*	0.359*	0.342*	0.300*	1

tobacco group 1 = 0 days; tobacco group 2 = 1-2 days; tobacco group 3 = 3-9days; tobacco group 4 = 10-29 days; tobacco group 5 = ≥30 days.

* indicates that there is a statistically significant difference between genders.

Table S5. Correlation matrix of tobacco use frequency groups by age, with significance indicators

	[tobacco group 1] * age	[tobacco group 2] * age	[tobacco group 3] * age	[tobacco group 4] * age	[tobacco group 5] * age
**[tobacco group 1] * age**	1				
**[tobacco group 2] * age**	0.724*	1			
**[tobacco group 3] * age**	0.605*	0.444*	1		
**[tobacco group 4] * age**	0.533*	0.375*	0.353*	1	
**[tobacco group 5] * age**	0.431*	0.312*	0.336*	0.186*	1

tobacco group 1 = 0 days; tobacco group 2 = 1-2 days; tobacco group 3 = 3-9days; tobacco group 4 = 10-29 days; tobacco group 5 = ≥30 days.

* indicates that there is a statistically significant difference between genders.

Table S6. Correlation matrix of alcohol use frequency groups by sex, with significance indicators

	**[alcohol group 1] * sex**	**[alcohol group 2] * sex**	**[alcohol group 3] * sex**	**[alcohol group 4] * sex**	**[alcohol group 5] * sex**
**[alcohol group 1] * sex**	1				
**[alcohol group 2] * sex**	0.833*	1			
**[alcohol group 3] * sex**	0.641*	0.601*	1		
**[alcohol group 4] * sex**	0.403*	0.433*	0.234*	1	
**[alcohol group 5] * sex**	0.386*	0.410*	0.264*	0.220*	1

alcohol group 1 = 0 days; alcohol group 2 = 1-2 days; alcohol group 3 = 3-9days; alcohol group 4 = 10-29 days; alcohol group 5 = ≥30 days.

* indicates that there is a statistically significant difference between genders.

Table S7. Correlation matrix of alcohol use frequency groups by age, with significance indicators

	[alcohol group 1] * age	[alcohol group 2] * age	[alcohol group 3] * age	[alcohol group 4] * age	[alcohol group 5] * age
**[alcohol group 1] * age**	1				
**[alcohol group 2] * age**	0.783*	1			
**[alcohol group 3] * age**	0.577*	0.516*	1		
**[alcohol group 4] * age**	0.362*	0.411*	0.266*	1	
**[alcohol group 5] * age**	0.285*	0.330*	0.169*	0.261*	1

alcohol group 1 = 0 days; alcohol group 2 = 1-2 days; alcohol group 3 = 3-9days; alcohol group 4 = 10-29 days; alcohol group 5 = ≥30 days.

* indicates that there is a statistically significant difference between genders.

Table S8. Association between tobacco use and stress-related sleep disturbance among adolescents by sex, age group, World Bank income group, and WHO region (excluding Tokelau and Afghanistan)

Group	Tobacco use days during the past 30 days, OR(95%CI)					*P* for trend
	0 days	1-2 days	3-9days	10-29 days	≥30 days	
**Total**	1.00	1.11(0.88-1.39)	1.62(1.23-2.12)	1.76(1.18-2.64)	1.98(1.39-2.81)	<0.001
**Sex**						
Males	1.00	0.94(0.66-1.35)	1.53(1.05-2.25)	1.74(1.01-3.02)	1.94(1.25-3.01)	0.007
Females	1.00	1.40(1.08-1.83)	1.83(1.28-2.61)	2.03(1.22-3.36)	2.59(1.58-4.25)	<0.001
**Age group**						
12-14 years	1.00	1.19(0.85-1.65)	1.59(0.94-2.69)	1.90(1.26-2.86)	2.71(1.45-5.08)	<0.001
15-17 years	1.00	1.06(0.79-1.42)	1.65(1.22-2.22)	1.72(0.99-2.98)	1.68(1.14-2.46)	0.016
**World Bank income group**						
Low income	1.00	1.53(0.91-2.55)	1.89(1.07-3.34)	1.94(0.90-4.19)	4.29(1.80-10.21)	0.022
Lower-middle income	1.00	0.88(0.60-1.30)	1.48(0.95-2.31)	1.64(0.86-3.14)	1.46(0.85-2.52)	0.126
Upper-middle income	1.00	1.35(1.02-1.78)	1.58(1.19-2.10)	1.83(1.23-2.72)	2.33(1.55-3.49)	<0.001
High income	1.00	1.07(0.84-1.36)	1.64(1.30-2.06)	1.65(1.18-2.29)	1.36(0.98-1.87)	<0.001
**WHO region**						
Africa	1.00	1.18(0.76-1.83)	1.52(0.97-2.36)	2.11(1.34-3.34)	1.55(0.82-2.93)	0.032
Americas	1.00	1.29(0.92-1.81)	1.39(0.97-1.98)	1.56(0.84-2.87)	3.16(1.72-5.81)	0.013
Eastern Mediterranean	1.00	1.72(1.34-2.20)	2.06(1.52-2.79)	1.86(1.27-2.73)	2.65(1.88-3.72)	<0.001
South-East Asia	1.00	1.03(0.56-1.90)	2.54(1.43-4.50)	1.98(0.87-4.51)	2.46(1.25-4.82)	0.023
Western Pacific	1.00	1.01(0.73-1.39)	0.97(0.57-1.63)	1.19(0.74-1.92)	1.57(1.01-2.44)	0.364

Adjusted for sex, age, survey year, intake of fast food, soft drinks, and fruit/vegetables, and World Bank income in the survey year for each country, days active 60 min plus past 7 days, PPP, alcohol use, region, how many days bullied past 30 days,

Table S9. Association between alcohol use and stress-related sleep disturbance among adolescents by sex, age group, World Bank income group, and WHO region (excluding Tokelau and Afghanistan)

Group	Alcohol use days during the past 30 days, OR(95%CI)					*P* for trend
	0 days	1-2 days	3-9days	10-29 days	≥30 days	
**Total**	1.00	1.53(1.31-1.78)	1.85(1.44-2.38)	2.21(1.44-3.39)	3.13(1.99-4.90)	<0.001
**Sex**						
Males	1.00	1.51(1.16-1.96)	1.66(1.10-2.51)	2.75(1.54-4.93)	3.10(1.75-5.48)	<0.001
Females	1.00	1.50(1.27-1.77)	2.02(1.54-2.65)	1.51(0.80-2.83)	3.18(1.46-6.95)	<0.001
**Age group**						
12-14 years	1.00	1.58(1.18-2.12)	1.87(1.22-2.85)	1.75(0.70-4.35)	4.02(1.86-8.67)	<0.001
15-17 years	1.00	1.52(1.27-1.82)	1.89(1.40-2.56)	2.49(1.63-3.81)	2.95(1.71-5.06)	<0.001
**World Bank income group**						
Low income	1.00	1.36(0.96-1.93)	1.42(0.84-2.40)	1.89(0.87-4.10)	6.16(2.04-18.60)	0.008
Lower-middle income	1.00	1.52(1.14-2.02)	2.39(1.49-3.81)	2.66(1.16-6.14)	3.74(2.07-6.77)	<0.001
Upper-middle income	1.00	1.45(1.20-1.76)	1.51(1.18-1.92)	1.96(1.16-3.31)	1.76(0.81-3.82)	<0.001
High income	1.00	1.35(1.16-1.57)	1.75(1.46-2.10)	1.59(1.19-2.13)	1.86(1.08-3.21)	<0.001
**WHO region**						
Africa	1.00	1.24(0.92-1.69)	1.72(1.12-2.64)	2.13(1.31-3.45)	4.73(2.31-9.70)	<0.001
Americas	1.00	1.47(1.19-1.83)	1.50(1.14-1.98)	2.51(1.44-4.38)	1.73(0.79-3.81)	0.002
Eastern Mediterranean	1.00	0.95(0.67-1.34)	0.84(0.55-1.27)	1.38(0.71-2.68)	2.43(0.92-6.44)	0.240
South-East Asia	1.00	1.76(1.04-2.97)	2.46(1.22-4.96)	1.58(0.42-5.94)	2.35(0.66-8.39)	0.034
Western Pacific	1.00	1.20(1.00-1.45)	1.44(0.99-2.11)	2.38(1.27-4.45)	2.84(1.44-5.58)	0.003

Adjusted for sex, age, survey year, intake of fast food, soft drinks, and fruit/vegetables, and World Bank income in the survey year for each country, days active 60 min plus past 7 days, PPP, alcohol use, region, how many days bullied past 30 days,

Table S10. Association between tobacco use and stress-related sleep disturbance among adolescents by sex, age group, World Bank income group, and WHO region (responses of "sometimes", "most of the time", and “always” were all defined as stress-related sleep disturbance)

Group	Tobacco use days during the past 30 days, OR(95%CI)					*P* for trend
	0 days	1-2 days	3-9days	10-29 days	≥30 days	
**Total**	1.00	1.01(0.74-1.39)	1.41(1.14-1.73)	1.05(0.73-1.51)	1.28(0.93-1.76)	0.012
**Sex**						
Males	1.00	0.93(0.61-1.41)	1.53(1.22-1.93)	1.04(0.65-1.67)	1.38(0.98-1.95)	0.001
Females	1.00	1.31(1.06-1.62)	1.22(0.86-1.73)	1.28(0.79-2.08)	1.34(0.80-2.25)	0.105
**Age group**						
12-14 years	1.00	1.25(1.00-1.57)	1.60(1.25-2.04)	1.57(1.15-2.12)	1.71(0.90-3.22)	<0.001
15-17 years	1.00	0.89(0.58-1.35)	1.31(0.99-1.73)	0.95(0.60-1.52)	1.20(0.87-1.67)	0.186
**World Bank income group**						
Low income	1.00	1.13(0.80-1.59)	1.35(0.89-2.04)	1.96(1.10-3.50)	2.65(1.12-6.23)	0.047
Lower-middle income	1.00	0.91(0.57-1.47)	1.44(1.03-2.01)	0.86(0.52-1.43)	1.14(0.69-1.87)	0.176
Upper-middle income	1.00	1.27(1.03-1.57)	1.53(1.26-1.85)	1.56(1.11-2.19)	1.61(1.24-2.08)	<0.001
High income	1.00	1.21(1.03-1.43)	1.57(1.32-1.86)	1.56(1.20-2.02)	1.12(0.88-1.44)	<0.001
**WHO region**						
Africa	1.00	1.29(0.87-1.89)	1.15(0.79-1.69)	1.70(1.07-2.70)	0.83(0.34-1.99)	0.024
Americas	1.00	1.30(1.04-1.62)	1.58(1.24-2.00)	1.11(0.68-1.82)	1.61(1.00-2.58)	<0.001
Eastern Mediterranean	1.00	1.55(1.29-1.88)	1.76(1.37-2.25)	1.57(1.16-2.13)	2.17(1.63-2.91)	<0.001
South-East Asia	1.00	0.81(0.38-1.71)	1.81(1.21-2.70)	0.90(0.50-1.62)	1.45(0.87-2.40)	0.008
Western Pacific	1.00	1.14(0.94-1.38)	1.15(0.84-1.56)	0.93(0.65-1.33)	1.24(0.88-1.74)	0.518

Adjusted for sex, age, survey year, intake of fast food, soft drinks, and fruit/vegetables, and World Bank income in the survey year for each country, how many days bullied past 30 days, days active 60 min plus past 7 days, PPP, alcohol use, region

Table S11. Association between alcohol use and stress-related sleep disturbance among adolescents by sex, age group, World Bank income group, and WHO region (responses of "sometimes", "most of the time", and “always” were all defined as stress-related sleep disturbance)

Group	Alcohol use days during the past 30 days, OR(95%CI)					*P* for trend
	0 days	1-2 days	3-9days	10-29 days	≥30 days	
**Total**	1.00	1.42(1.29-1.56)	1.51(1.30-1.76)	1.67(1.26-2.22)	1.74(1.14-2.68)	<0.001
**Sex**						
Males	1.00	1.38(1.19-1.59)	1.38(1.12-1.70)	1.87(1.27-2.74)	1.90(1.19-3.04)	<0.001
Females	1.00	1.47(1.30-1.66)	1.76(1.43-2.18)	1.31(0.86-1.98)	1.45(0.68-3.09)	<0.001
**Age group**						
12-14 years	1.00	1.36(1.17-1.59)	1.38(1.06-1.79)	0.99(0.59-1.67)	1.48(0.69-3.19)	0.001
15-17 years	1.00	1.50(1.34-1.67)	1.65(1.39-1.96)	2.19(1.64-2.92)	2.00(1.21-3.29)	<0.001
**World Bank income group**						
Low income	1.00	1.51(1.19-1.93)	1.55(1.12-2.16)	1.83(0.95-3.49)	4.47(1.29-15.57)	<0.001
Lower-middle income	1.00	1.41(1.20-1.66)	1.26(0.91-1.73)	1.38(0.78-2.45)	1.47(0.77-2.81)	0.001
Upper-middle income	1.00	1.41(1.26-1.58)	1.76(1.50-2.06)	1.89(1.35-2.63)	1.24(0.60-2.57)	<0.001
High income	1.00	1.44(1.31-1.58)	1.62(1.44-1.83)	1.60(1.26-2.03)	1.46(0.87-2.47)	<0.001
**WHO region**						
Africa	1.00	1.61(1.31-1.98)	1.44(1.09-1.92)	1.97(1.17-3.32)	2.94(1.35-6.42)	<0.001
Americas	1.00	1.54(1.35-1.76)	1.90(1.58-2.30)	2.20(1.51-3.20)	1.58(0.72-3.45)	<0.001
Eastern Mediterranean	1.00	0.84(0.65-1.09)	0.84(0.62-1.15)	0.75(0.43-1.32)	0.93(0.37-2.32)	0.532
South-East Asia	1.00	1.36(1.06-1.75)	1.52(1.01-2.29)	1.11(0.55-2.23)	0.69(0.21-2.28)	0.059
Western Pacific	1.00	1.34(1.17-1.52)	1.25(1.02-1.53)	1.42(0.87-2.34)	1.36(0.72-2.57)	<0.001

Adjusted for sex, age, survey year, intake of fast food, soft drinks, and fruit/vegetables, and World Bank income in the survey year for each country, how many days bullied past 30 days, days active 60 min plus past 7 days, PPP, tobacco use, region
